# At the crossroads of immunity and hijacking: dual roles of DDX proteins during host–virus interactions

**DOI:** 10.3389/fcimb.2026.1958960

**Published:** 2026-09-30

**Authors:** Tingxuan Xu, Shengyu Wang, Yongchang Zhou, Xin Sun, Hong Song

**Affiliations:** 1 Key Laboratory of Infectious Diseases and Biosafety, Education Department of Guizhou Province, Zunyi, China; 2 Department of Microbiology, School of Preclinical Medicine, Zunyi Medical University, Zunyi, China

**Keywords:** DDX proteins, DNA viruses, innate immunity, RNA viruses, viral infection

## Abstract

The DEAD-box (DDX) protein family, constituting a critical subgroup of the Superfamily 2 (SF2) helicase family, plays extensive roles in nucleic acid metabolism, gene expression regulation and innate immune signaling, and exerts a key regulatory role during viral infection. During viral infection, DDX proteins function as both RNA helicases and host restriction factors, exerting antiviral effects by recognizing viral nucleic acids, regulating signaling pathways and maintaining the host immune response. Conversely, through complex and diverse mechanisms, viruses can hijack or antagonize DDX proteins to facilitate their replication, transcription, translation and immune evasion. Focusing on the DDX protein family, this review systematically elucidates their molecular mechanisms of action across the life cycles of various viruses. Specifically, it examines the regulation of viral nucleic acid metabolism by DDX proteins, their influence on innate immune signaling pathways, and the molecular strategies employed by viruses to antagonize DDX proteins, while synthesizing the functional similarities and differences among members of the DDX protein family across distinct viral infections. This review aims to establish a theoretical framework for the in-depth analysis of virus–host interactions and the discovery of novel antiviral targets.

## 1 Introduction

The DEAD-box (DDX) protein family comprises a large and diverse group of highly conserved RNA helicases. The family derives its name from the conserved aspartic acid–glutamic acid–alanine–aspartic acid (Asp-Glu-Ala-Asp, DEAD) sequence within the Walker B motif of its core domain. This core domain comprises two RecA modules, each possessing ATP-binding and RNA-binding activities, and contains nine highly conserved sequence motifs. It is flanked by the N-terminal and C-terminal domains, which exhibit greater sequence diversity (Hirth et al., 2024; Tapescu and Cherry, 2024) and mediate key biological functions (Tanner et al., 2003; Song et al., 2023) (**Figure 1**). As central regulatory factors in RNA metabolism, DDX proteins are involved in several critical processes, including transcription, translation initiation, and messenger ribonucleic acid (mRNA) transport. Some DDX proteins can also interact with protein kinases; for example, by regulating the catalytic activity of microtubule affinity-regulating kinase 1 (MARK1), they participate in downstream signaling pathways (Chao et al., 2006; Soto-Rifo et al., 2012; Xing et al., 2019; Han et al., 2020; Yang et al., 2023; Hirth et al., 2024).

**Figure 1:**
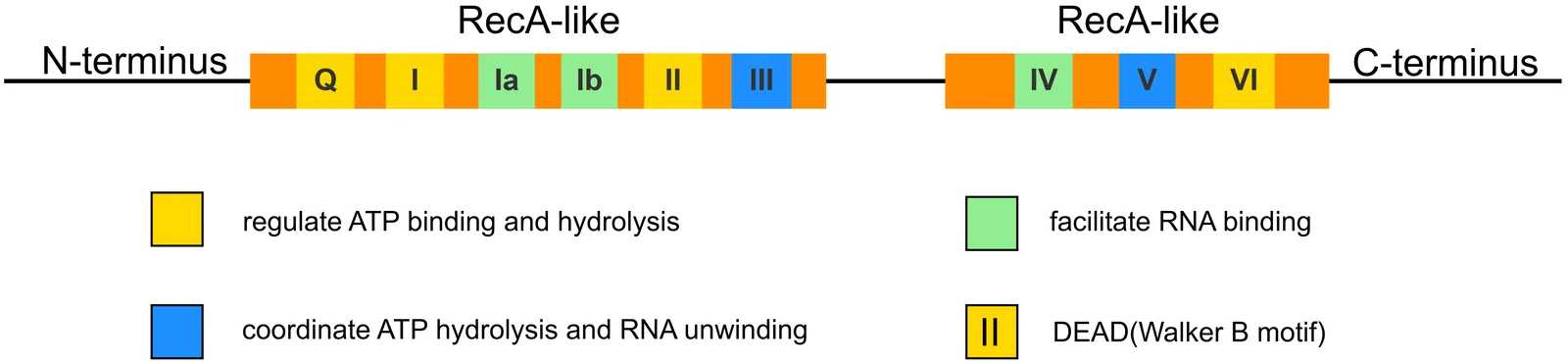
Core domain of the DDX protein.

During pathogen infection, DDX proteins act as host factors, participating in the intricate interactions between the host and the pathogen. Given this critical role, elucidating the interaction mechanisms between DDX proteins and viral pathogens is essential for understanding viral pathogenesis. These mechanistic insights represent a promising avenue for developing novel therapeutic strategies against viral pathogens. As strictly intracellular parasites, viruses utilize the host cell’s intrinsic molecules to carry out their life cycle and evade the host’s immune response. In this context, DDX proteins utilize their conserved catalytic cores to recognize RNA structural features in a sequence-independent manner (Tapescu et al., 2023; Tapescu and Cherry, 2024), thereby contributing to intracellular viral RNA unwinding, replication, transcription, translation, and assembly of viral particles (Linder and Jankowsky, 2011; Paine et al., 2019). Additionally, they regulate the host’s innate immune response by modulating relevant signaling pathways and protein expression (Ma et al., 2025a; Mao et al., 2024; Szappanos et al., 2018; Sandhu and Damania, 2025; Wu et al., 2026), thereby controlling viral infection. In the innate immune response, certain DDX proteins act as pattern recognition receptors (PRRs) to recognize viral nucleic acids and subsequently activate relevant signaling pathways (Miyashita et al., 2011; Zhang et al., 2011b, 2011a; Parthun et al., 2025). For example, DDX50 activates the Interferon regulatory factor 3 (IRF3) signaling pathway upon stimulation by viral RNA or during infection with RNA and DNA viruses (Pallett et al., 2022). Furthermore, the DDX1-DDX21-DHX36 complex recognizes double-stranded RNA (dsRNA) and utilizes the TIR domain-containing adaptor-inducing interferon-β (TRIF) pathway to activate the Type I Interferons (IFN-I) response (Zhang et al., 2011a), among other mechanisms. Moreover, in immune signaling cascades, DDX proteins not only act as sensors but also function as scaffold molecules to exert regulatory effects. For example, DDX3X, acting as a scaffold protein, directly facilitates the phosphorylation of IRF3 by IκB kinase-ϵ (IKKϵ), thereby contributing to the activation and regulation of the IRF3 pathway (Gu et al., 2013). Whether acting as PRRs or scaffold proteins, DDX proteins play a crucial role in regulating the host’s innate immune response.

DDX proteins are not only key players in the innate immune system but also serve as vital tools for viruses to facilitate replication, reactivation, and immune evasion. Viruses can hijack DDX proteins to enhance viral replication or evade host immune surveillance, thereby enabling persistent infection or latent reactivation (Barra et al., 2025; Dong et al., 2025; Chen et al., 2026). For example, the Human papillomavirus regulatory protein E2 (HPV E2) hijacks DDX11 (ChlR1) to facilitate the binding of its own mitotic chromatin (Harris et al., 2016); the hepatitis C virus (HCV) hijacks DDX6 and the Processing body (P-body) to create conducive conditions for its own replication (Ariumi et al., 2011). It is precisely this dual role of DDX proteins in viral regulation that makes them a key battleground in the interplay between virus and host.

DDX proteins and viruses share a complex, multifaceted relationship characterized by mutual influence and restraint, which is of great significance for the onset, progression, treatment and prevention of infectious diseases. Therefore, an in-depth investigation of the interactions between DDX proteins and viruses and their molecular mechanisms paves the way for the prevention and treatment of viral infectious diseases. This review will be organized primarily according to the type of viral nucleic acid, covering a range of viruses including hepatitis viruses, arboviruses, herpesviruses and oncoviruses. It will systematically summarize the mechanisms of their interactions with DDX proteins and the associated immune signaling pathways, and elucidate specific virus-DDX protein interactions, thereby offering novel theoretical insights into the prevention and control of viral infections.

## 2 DNA virus infection and DDX protein functions

After a host cell becomes infected, DNA viruses primarily rely on DNA polymerases to catalyze deoxyribonucleotide polymerization reactions. They also utilize enzymes encoded by either the host or the virus itself to carry out essential processes such as genome unwinding, primer synthesis, and damage repair (Gassmann et al., 1988; Tian et al., 2009; Lawler et al., 2024), with members of the DDX protein family playing a notable role in these activities. Viruses exploit DDX proteins to facilitate their own replication or to evade the host immune response. At the same time, upon detecting viral DNA, the host can activate classical signaling pathways like cyclic GMP-AMP synthase–stimulator of interferon genes (cGAS-STING), Toll-like receptor 9 (TLR9), and Retinoic acid-inducible gene I–mitochondrial antiviral signaling protein (RIG-I-MAVS) to initiate an innate immune response (Ablasser et al., 2009; Fiola et al., 2010; Civril et al., 2013; Dansako et al., 2016; Hristova et al., 2024). In this context, DDX proteins act as pattern recognition receptors (PRRs), recognizing viral DNA and subsequently activating downstream signaling pathways, thereby triggering the host’s innate immune defenses. Given the differences in genomic structure, replication sites, and host exploitation strategies among DNA viruses, they have evolved diverse replication approaches. Moreover, as key host regulatory factors, DDX proteins are extensively involved in both viral infection and the host immune response, performing distinct functions throughout the life cycles of different DNA viruses and in the activation of various signaling pathways. The following sections will discuss several DNA viruses individually.

### 2.1 Hepatitis B virus

The hepatitis B virus (HBV) primarily infects the liver, leading to hepatitis, and its replication cycle is critically dependent on the formation of covalently closed circular DNA (cccDNA) within the cell nucleus (Wei and Ploss, 2020; Gómez-Moreno and Ploss, 2024). Additionally, HBV exhibits unique spatiotemporal dynamics, leading to distinct mechanisms through which DDX proteins mediate its replication compared to other hepatitis viruses. During the transcription of HBV cccDNA, DDX5 competitively binds to the E3 ligase mex-3 RNA binding family member B (Mex3b) in the presence of HOX Transcript Antisense Intergenic RNA (HOTAIR), thereby silencing cccDNA transcription by modulating the stability of the polycomb repressive complex 2 (PRC2) subunit SUZ12 complex (Zhang et al., 2016). In contrast, DDX3X directly inhibits cccDNA transcription through a mechanism independent of interactions with HBV polymerase (HBV Pol) and Hepatitis B virus X protein (HBx) (Ko et al., 2014). Furthermore, DDX5 and DDX17 serve as key regulatory factors that govern the heterogeneity of HBV transcription termination. Specifically, they reduce the stability of HBV RNA by inhibiting the recognition of the cytoplasmic polyadenylation signal (cPAS), thereby suppressing efficient cccDNA transcription and HBV replication (Chapus et al., 2024). Moreover, DDX17 acts as an endogenous host restriction factor; through its enzymatic activity, it competitively binds to the 5’epsilon motif of HBV polymerase, displacing pregenomic RNA (pgRNA), thus interfering with the pgRNA capsidization process (Mao et al., 2021) and further suppressing HBV. During the reverse transcription process, DDX3X binds to HBV Pol, thereby suppressing HBV reverse transcription and subsequent viral DNA synthesis (Wang et al., 2009). DDX proteins play a dual role in HBV replication: on one hand, they inhibit HBV replication by interfering with its transcription and reverse transcription processes; on the other hand, they can be co-opted by HBV to regulate its own replication. For instance, HBV Pol binds to DDX3X, disrupting its interaction with TANK-binding kinase 1 (TBK1)/IKKϵ, thereby inhibiting the IRF signaling pathway and enabling the virus to evade the host’s innate immune response (Wang and Ryu, 2010; Yu et al., 2010). Similarly, DDX17 expression can be up-regulated by HBx, which in turn promotes HBV transcription and replication; reciprocally, DDX17 can also enhance HBx expression by stimulating the HBx promoter (Dong et al., 2022). Additionally, DDX proteins can directly activate host signaling pathways to trigger antiviral immune responses. For example, DDX5 enhances the activity of the interferon (IFN) signaling pathway by promoting Signal transducer and activator of transcription 1 (STAT1) translation (Sun et al., 2022), thereby interfering with HBV replication.

### 2.2 Herpesviruses

Herpesviruses exhibit a distinctive replication strategy that involves three key phases: the lytic cycle, latency, and reactivation. Lytic replication occurs when the virus completes its replication cycle inside a host cell and then lyses the cell to release newly formed viral particles. The transition from a latent state to active replication is termed lytic reactivation. This process plays a central role in the persistence of chronic herpesvirus infections, as well as the recurrence and progression of associated diseases (Traylen et al., 2011; Liu et al., 2023). DDX proteins modulate the progression of viral infection by participating in these replication dynamics. In the context of γ-herpesviruses, DDX24 and DDX49 can exert antiviral effects during lytic reactivation by recognizing viral transcripts and subsequently inducing the production of IFN-I (Serfecz et al., 2022). Conversely, DDX5 and DDX17 function as essential host factors that interact with viral protein kinases, such as viral protein kinase (vPK) encoded by Kaposi’s sarcoma-associated herpesvirus (KSHV, also known as HHV-8) and Epstein-Barr virus (EBV) protein kinase BGLF4 (BGLF4). In the absence of these two factors, the level of viral lytic reactivation is significantly reduced (Sandhu and Damania, 2025). Additionally, DDX5 and DDX17 may regulate EBV replication through alternative mechanisms. For example, DDX5 can epigenetically regulate interferon-induced transmembrane protein 1 (IFITM1), which in turn blocks EBV invasion of endothelial cells mediated by the Ephrin A-type receptor 2 (EphA2), thereby inhibiting viral latency and proliferation (Yang et al., 2024). DDX17, in contrast, is recruited by the YTH N6-methyladenosine RNA binding protein 1 (YTHDF1) into an RNA degradation complex during gene expression. This recruitment accelerates the de-capping and degradation of EBV RNA, thereby downregulating the expression of EBV genes at the post-transcriptional level (Xia et al., 2021). While DDX5 and DDX17 are homologous proteins, their respective mechanisms for interfering with EBV replication are entirely distinct, underscoring the unique nature of DDX proteins in the regulation of viral replication. Moreover, DDX3X also regulates herpesvirus replication. DDX3X not only directly interacts with the virus-encoded nuclear egress complex at the nuclear envelope but also binds to the Herpes simplex virus US3 protein (pUs3), thereby promoting the incorporation of pUs3 into mature viral particles. pUs3 facilitates the transit of viral particles across the outer nuclear membrane, a critical step in nuclear egress (Khadivjam et al., 2023), which subsequently promotes the reactivation of latent herpesvirus.

While DDX proteins play a key regulatory role in herpesvirus infection, herpesviruses have also evolved strategies to hijack these same proteins to enhance their own replication and gene expression, as demonstrated by herpes simplex virus (HSV). Specifically, HSV-1 can suppress DDX41 by directly targeting the 3’ untranslated region (3’UTR) of its mRNA through a virally encoded microRNA (miRNA), thereby inhibiting DDX41 translation and protein expression. This suppression subsequently blocks the activation of the IFN-I pathway, leading to a reduced expression of interferon-β (IFN-β) and its downstream interferon-stimulated gene, Myxovirus resistance protein 1 (Mx1), which ultimately promotes viral replication (Duan et al., 2019). In contrast, DDX20 facilitates the interaction between TBK1 and IRF3, thereby enhancing IRF3 phosphorylation triggered by external stimuli, which ultimately promotes IFN-β expression. This increase in IFN-β further upregulates the expression of interferon-stimulated genes (ISGs), thus exerting a potent antiviral effect (Chen et al., 2024). Further studies have revealed that HSV-1 can incorporate DDX3X into mature viral particles, ensuring optimal viral infectivity; moreover, viral gene expression, genomic replication, and the production of progeny viruses all depend on sufficient levels of DDX3X protein expression within the host cell (Khadivjam et al., 2017). The utilization of DDX3X is not limited to HSV-1 but extends to HSV-2, which incorporates DDX3X into assembling viral particles to regulate the nuclear export of newly formed viral particles, thereby facilitating the critical transition from intranuclear assembly to intracellular release. As large numbers of DDX3X-carrying viral particles are released, DDX3X becomes significantly depleted within the host cell; this not only enhances viral nuclear export but also indirectly inhibits the host cell’s normal physiological functions, thereby creating a more favorable microenvironment for HSV-2 replication (Piazza et al., 2025).

Similarly, human cytomegalovirus (HCMV), which belongs to the herpesvirus family, is also regulated by DDX proteins. For instance, DDX3X binds to the protein encoded by the viral m139 gene, thereby suppressing IFN-I production and disrupting DDX3X-dependent RNA metabolism within host cells (Puhach et al., 2020). In the later stages of infection, HCMV optimizes the interaction interface between the viral protein Human cytomegalovirus UL69 protein (Pul69) and DDX39B/DDX39A (UAP56/URH49); this optimization enhances competitive binding efficiency, facilitates the preferential transport of viral mRNA (Lischka et al., 2006; Thomas et al., 2020), and ultimately promotes viral replication. Additional research has revealed that knocking down DDX21 results in the accumulation of R-loops, which impedes the transcription of late viral genes and consequently inhibits HCMV replication (Hao et al., 2019). Overall, DDX proteins play essential roles throughout every stage of HCMV infection.

These findings demonstrate that DDX proteins exert a profound regulatory impact on herpesviruses across γ-herpesviruses, HSV, and HCMV. These effects encompass not only viral replication but also the activation of host signaling pathways, thereby underscoring the critical role of DDX proteins in the herpesvirus infectious cycle.

### 2.3 Human papillomavirus

As a significant oncovirus, Human papillomavirus (HPV) relies on DDX proteins to regulate its life cycle and genomic replication, often hijacking these proteins to establish persistent infection. Previous studies have demonstrated that the viral E2 protein can specifically bind to DDX11, also known as ChlR1. When DDX11 is recruited to replication stress sites, it can, via protein-protein interactions, localize the E2-HPV genome complex to the replication fork, reduce the binding of E2 to the nuclear matrix, and promote the pre-mitotic loading of the viral genome onto the host cell chromatin. This ultimately facilitates the formation and maintenance of high-copy HPV episomes (Harris et al., 2016), thereby sustaining persistent viral infection and promoting cellular carcinogenesis. Conversely, some DDX proteins may also exert antiviral effects. For example, DDX48 (eIF4a3) can enhance the efficiency of the eukaryotic initiation factor 4A3-nonsense-mediated mRNA decay (eIF4a3-NMD) pathway and degrade human papillomavirus early oncoproteins E6 and E7 mRNA, thereby inhibiting E7 protein production and suppressing HPV gene expression (Meznad et al., 2021), which constitutes a negative regulatory mechanism for HPV replication. Furthermore, some studies indicate that DDX proteins may also influence tumor development (Li et al., 2026), thereby highlighting their importance in regulating both oncoviral infection and post-tumor-initiation processes *in vivo* (Cai et al., 2025). Collectively, the dual role of DDX proteins—both supporting and suppressing HPV activity—underscores their complex involvement in viral pathogenesis and cancer progression, making them critical targets for further research into therapeutic interventions.

In summary, DDX proteins exert multifaceted and bidirectional regulatory effects during DNA viral infection (**Table 1**). They are extensively involved in critical stages of the viral life cycle, including replication, transcription, nuclear export, and nucleocapsid assembly, and concurrently modulating the host’s innate immune response and the activation of key signaling pathways. Furthermore, during this process, DNA viruses exploit DDX proteins as molecular scaffolds or interaction partners to facilitate sustained expression of the viral genome, reactivation of latent infections, and immune evasion, thereby enabling persistent infection or reinfection. In light of the unique features of DNA viruses—such as their highly stable genomes, propensity for chronic infection, and resistance to eradication during latency—specifically targeting DDX proteins to disrupt virus-DDX interactions, inhibit DDX proteolytic activity, or modulate the activation of downstream immune signaling pathways presents a promising strategy for inhibiting viral replication while blocking latent reactivation. This approach offers a novel therapeutic avenue for managing persistent viral infections and reducing the risk of recurrence and malignant transformation associated with viral diseases.

**Table 1:** The relationship between DDX proteins and DNA viruses.

Role in viral infection	DDX proteins	Viruses	Reference
Promotion of viral replication	DDX5, DDX17	γ-herpesviruses	(Sandhu and Damania, 2025)
Inhibition of viral replication	DDX3X	HBV	(Wang et al., 2009)
	DDX5	HBV	(Zhang et al., 2016)
		EBV	(Yang et al., 2024)
	DDX17	HBV	(Chapus et al., 2024)
		EBV	(Xia et al., 2021)
	DDX21	HCMV	(Hao et al., 2019)
	DDX24	γ-herpesviruses	(Serfecz et al., 2022)
	DDX48	HPV	(Meznad et al., 2021)
	DDX49	γ-herpesviruses	(Serfecz et al., 2022)
DDX proteins exploited by viruses	DDX3X	HBV	(Wang and Ryu, 2010; Yu et al., 2010)
		HSV-2	(Piazza et al., 2025)
		HCMV	(Puhach et al., 2020)
	DDX11	HPV	(Harris et al., 2016)
	DDX17	HBV	(Dong et al., 2022)
	DDX21	HCMV	(Hao et al., 2019)
	DDX39A/DDX39B	HCMV	(Thomas et al., 2020)
	DDX41	HSV-1	(Duan et al., 2019)

## 3 RNA virus infection and DDX protein functions

As RNA helicases associated with the host’s innate immune response, DDX proteins are capable of recognizing viral RNA and modulating key processes such as viral replication and gene expression. Since the RNA polymerase responsible for RNA virus replication lacks proofreading activity, base mismatches frequently occur during replication, resulting in high rates of viral mutations (Steinhauer et al., 1992; Smith, 2017). Consequently, molecular signatures generated during RNA virus replication—including dsRNA replication intermediates and atypical RNA species—can be detected by the host’s innate immune receptors, such as RIG-I-like receptors (RLRs) and Toll-like receptors (TLRs). This recognition subsequently activates signaling pathways such as MAVS-TBK1-IRF3/7 and nuclear factor κ-light-chain-enhancer of activated B cells (NF-κB), triggering an IFN-I response and the secretion of pro-inflammatory factors, thereby inhibiting viral replication (Yu et al., 2023; Shrestha et al., 2024; Pinto et al., 2011). DDX proteins can indirectly influence viral replication by modulating key nodes within these signaling pathways. Therefore, a thorough investigation into the relationship between DDX proteins and RNA viruses will help address challenges such as drug and vaccine failure caused by viral mutations, and will provide new, more precise, broad-spectrum, and resilient to resistance strategies for the prevention and treatment of infectious diseases induced by RNA viruses. The specific cytokines, signaling pathways, and DDX proteins involved vary depending on the type of RNA virus; these will be briefly outlined below.

### 3.1 Respiratory viruses

The influenza virus, as a pathogen that causes seasonal illness and can be transmitted across species, has long been the subject of considerable attention. Based on antigenic differences in the nucleoprotein (NP) and matrix protein 1 (M1), influenza viruses can be classified into four types: A, B, C, and D (Liang, 2023), among which influenza A virus (IAV) is the most prevalent. Unlike other RNA viruses, influenza viruses undergo transcription and replication within the cell nucleus rather than in the cytoplasm (Chou et al., 2013); the mRNA produced within the nucleus must be transported to the cytoplasm to undergo translation (Plotch et al., 1981). DDX19 is critical for the transcription and nuclear export of IAV. IAV exploits the RNA-binding, nuclear pore complex protein NUP214-binding, and adenosine triphosphatase (ATPase) activities of DDX19 to facilitate the nuclear export of viral mRNA to the cytoplasm for translation. Any disruption to these DDX19 activities during this process inhibits viral mRNA transport and thereby hinders the IAV replication cycle (Diot et al., 2016). In addition, IAV can also interact with DDX21. Specifically, IAV binds directly to DDX21 through its non-structural protein 1 (NS1), disrupting DDX21’s interaction with viral ribonucleic acid (vRNA) and thereby suppressing its antiviral activity to sustain viral replication; conversely, DDX21 exerts antiviral activity by directly binding to the IAV polymerase basic protein 1 (PB1) protein and inhibiting its interaction with the Polymerase acidic protein (PA) and Polymerase basic protein 2 (PB2) polymerase subunits (Chen et al., 2014).

Severe acute respiratory syndrome coronavirus 2 (SARS-CoV-2), the pathogen responsible for coronavirus disease 2019 (COVID-19), is an RNA virus characterized by high transmissibility and mutation rates (Starr et al., 2022; Dadonaite et al., 2024; Ma et al., 2025b), and infection with this pathogen has also been associated with DDX proteins. Previous research has demonstrated that DDX42 can directly bind to SARS-CoV-2 RNA, thereby inhibiting the formation of the replication-transcription complex (RTC) and hindering viral RNA synthesis, ultimately suppressing viral replication (Bonaventure et al., 2022). In the context of viral replication, DDX21 limits viral proliferation and spread by interacting with the SARS-CoV-2 nucleocapsid protein (Ariumi, 2022). Additionally, SARS-CoV-2 can exploit DDX proteins to enhance its own replication; for instance, the viral nucleocapsid protein can hijack DDX6 to facilitate this process. Furthermore, DDX1 and DDX5 serve as essential host factors for SARS-CoV-2 replication, aiding in the completion of the viral replication cycle (Ariumi, 2022). The viral non-structural protein 1 (Nsp1) further promotes viral replication by increasing DDX5 expression through the activation of the T-cell nuclear factor (NFAT) signaling pathway (Lui et al., 2024).

### 3.2 Hepatitis C virus

In addition to respiratory viruses, HCV is similarly regulated by DDX proteins. Previous studies have demonstrated that DDX6 is essential for efficient HCV replication, and its helicase activity plays a critical role in HCV genome amplification (Jangra et al., 2010). Furthermore, DDX5 facilitates HCV replication and viral assembly by directly interacting with the HCV RNA-dependent RNA polymerase, Nonstructural protein 5B (NS5B) (Dutta et al., 2012). DDX3X further activates the inhibitor of nuclear factor κB kinase subunit α (IKK-α) signaling pathway through binding to the HCV 3’UTR, thereby promoting lipid droplet formation and viral assembly (Pène et al., 2015). Additionally, DDX6, as a key component of host P-bodies, can be hijacked by HCV and localized around lipid droplets, where it forms complexes with viral proteins in stress granules, creating a microenvironment that supports HCV replication and assembly (Ariumi et al., 2011). Concurrently, HCV can also exploit DDX proteins to evade the host immune response; for instance, through its core protein, it inhibits DDX3X-mediated translation of the protein activator of the interferon-induced protein kinase (PACT), or causes its relocalization to replication sites, thereby suppressing the IFN-I signaling pathway and facilitating viral evasion of host innate immunity (Lai et al., 2016). Furthermore, HCV can interact with the IKKϵ and suppress the expression of Linc-Pint, thereby blocking its binding to DDX24, attenuating the host immune response, and ultimately enabling the establishment of chronic infection through immune evasion (Lai et al., 2016; Khatun et al., 2021; Kang et al., 2022).

### 3.3 Mosquito-borne viruses

The Chikungunya virus (CHIKV), a significant mosquito-borne RNA virus, is regulated by DDX proteins throughout the infection cycle. Previous research has demonstrated that the DDX42 helicase suppresses CHIKV propagation by binding to Long Interspersed Nuclear Element 1 (LINE-1) RNA, thereby mediating a broad-spectrum antiviral effect (Bonaventure et al., 2022). Similarly, DDX56 recognizes and binds to CHIKV RNA, promoting its destabilization and subsequent degradation, which effectively inhibits viral infection and replication (Taschuk et al., 2020). Additionally, during CHIKV replication, DDX39A recognizes and binds to highly conserved secondary RNA structures within the CHIKV genome, disrupting the assembly of the replication complex and consequently inhibiting CHIKV RNA synthesis (Tapescu et al., 2023). Thus, DDX proteins modulate CHIKV replication through mechanisms such as structural recognition and enzymatic activity.

As another mosquito-borne RNA virus, dengue virus (DENV) has garnered significant attention due to its extensive global distribution and high incidence rates. Current research indicates that specific DDX proteins are critical regulators of DENV infection and replication during the early stages of the viral life cycle by influencing viral RNA, cellular signaling pathways, and the interferon response. Among these, DDX6 acts as a key host factor that interacts with DENV RNA, specifically binding to the Dumbbell structure 1 (DB1) and Dumbbell structure 2 (DB2) stem-loop structures in the 3’UTR of DENV-2 to facilitate viral RNA translation and replication complex assembly, thereby stabilizing the viral RNA structure (Ward et al., 2011). Additionally, DDX17 promotes viral replication by binding to viral double-stranded RNA, a mechanism that inhibits both interferon promoter activity and stress granule formation (Han et al., 2025). DDX25 enhances viral infection by interfering with the activation of IRF3 and NF-κB through the RIG-I signaling pathway, ultimately blocking the production of IFN-β (Feng et al., 2017). In contrast, DDX3X promotes IFN-β transcription by activating the IRF3 and NF-κB pathways, thereby initiating the IFN-I antiviral response and inhibiting viral infection. However, the DENV capsid protein binds to and downregulates DDX3X expression, allowing the virus to evade host immune surveillance (Li et al., 2015).

Another significant mosquito-borne virus is the epidemic type B encephalitis virus, commonly referred to as Japanese encephalitis virus (JEV). During its replication cycle, JEV modulates the expression of DDX proteins to enhance its own propagation; conversely, specific DDX proteins can either promote or inhibit viral replication in a context-dependent manner. JEV interacts with cellular miR-1-3p through JEV-induced non-coding RNA 1 (JINR1), thereby upregulating DDX5 expression and subsequently promoting viral replication (Tripathi et al., 2025). Additionally, DDX5 specifically binds to the JEV 3’UTR and directly interacts with the JEV core protein, nonstructural protein 3 (NS3), and nonstructural protein 5 (NS5), via its helicase activity to facilitate viral RNA replication (Li et al., 2013). Furthermore, during JEV infection, DDX3X facilitates viral replication by co-localizing with the 5’and 3’untranslated regions of the virus (Li et al., 2014, 2025; Nelson et al., 2021); it also induces the expression of IFN-β1 and the chemokines C-C motif chemokine ligand 5 (CCL5, also known as RANTES) thereby contributing to the host’s innate antiviral immune response (Lai et al., 2016). At the same time, the JEV nonstructural protein (NS1) can directly bind to DDX3X, thereby suppressing the expression of IFN-β and ISGs and evading the host’s innate immune response; conversely, JEV infection can also induce the phosphorylation of DDX3X, thereby inhibiting viral replication (Dong et al., 2025).

Beyond its activity against CHIKV, DENV, and JEV, the broad-spectrum antiviral protein DDX42 also effectively suppresses Zika virus (ZIKV). Specifically, DDX42 directly targets the ZIKV ribonucleoprotein complex, leading to the inhibition of viral replication (Bonaventure et al., 2022).

### 3.4 Enterovirus

Furthermore, enterovirus (EV) infections are tightly regulated by DDX proteins. Notably, enterovirus 71 (EV71), the primary pathogen responsible for hand, foot, and mouth disease, has caused numerous large-scale outbreaks, thereby garnering substantial research interest. DDX proteins play pivotal roles in EV71 replication. For instance, the key host factor DDX6 exhibits a dual function: it serves as a scaffold protein for P-bodies, where it interacts with viral RNA to facilitate replication (Fan et al., 2021); in contrast, it binds EV71 RNA to form an RNA-protein complex that promotes the RIG-I-mediated interferon response, thereby inhibiting viral replication (Zhang et al., 2021). This dual role underscores the functional versatility of the DDX protein family.

### 3.5 Other related RNA viruses

Furthermore, accumulating evidence indicates that DDX17 effectively inhibits the replication of rabies virus (RABV) via a G protein-dependent mechanism (Zhang et al., 2024b); similarly, both DDX21 and DDX60 suppress the replication of hantaviruses via an interferon-dependent mechanism (Ma et al., 2017), while DDX33 and DDX58 restrain the replication of human rhinovirus (hRV) by upregulating NOD-like receptor family pyrin domain-containing 3 (NLRP3) and interleukin-1β (IL-1β) (Liu et al., 2019).

Collectively, these studies indicate that the DDX protein family plays a pivotal role in regulating RNA viral infection and replication, through mechanisms such as binding to viral RNA structures, modulating viral RNA integrity via enzymatic activity, competitively interacting with cellular factors, and participating in the host’s innate immune response. Furthermore, some DDX proteins exhibit dual functionalities (**Table 2**), which underscores the complexity of their functions during viral infection and offers new insights into the mechanisms of pathogenesis and the development of potential therapeutic strategies.

**Table 2:** The relationship between DDX proteins and RNA viruses.

Role in viral infection	DDX proteins	Viruses	Reference
Promotion of viral replication	DDX3X	HCV	(Pène et al., 2015)
		JEV	(Li et al., 2014, 2025; Nelson et al., 2021)
	DDX5	SARS-CoV-2	(Lui et al., 2024)
		HCV	(Dutta et al., 2012)
		JEV	(Li et al., 2013)
	DDX6	HCV	(Jangra et al., 2010; Ariumi et al., 2011)
		DENV	(Ward et al., 2011)
		EV71	(Fan et al., 2021)
	DDX17	DENV	(Han et al., 2025)
	DDX19	IAV	(Diot et al., 2016)
	DDX25	DENV	(Feng et al., 2017)
Inhibition of viral replication	DDX3X	DENV	(Li et al., 2015)
		JEV	(Lai et al., 2016)
	DDX6	EV71	(Zhang et al., 2021)
	DDX17	RABV	(Zhang et al., 2024b)
	DDX21	IAV	(Chen et al., 2014)
		SARS-CoV-2	(Ariumi, 2022)
		Hantavirus	(Ma et al., 2017)
	DDX33	hRV	(Liu et al., 2019)
	DDX39A	CHIKV	(Tapescu et al., 2023)
	DDX42	SARS-CoV-2	(Bonaventure et al., 2022)
		CHIKV	(Bonaventure et al., 2022)
		ZIKV	(Bonaventure et al., 2022)
	DDX56	CHIKV	(Taschuk et al., 2020)
	DDX58	hRV	(Liu et al., 2019)
	DDX60	Hantavirus	(Ma et al., 2017)
DDX proteins exploited by viruses	DDX3X	HCV	(Lai et al., 2016)
		DENV	(Li et al., 2015)
		JEV	(Dong et al., 2025)
	DDX5	JEV	(Tripathi et al., 2025)
	DDX21	IAV	(Chen et al., 2014)
	DDX24	HCV	(Lai et al., 2016; Khatun et al., 2021; Kang et al., 2022)

## 4 Retrovirus infection and the DDX protein functions

Retroviruses are a distinctive class of viruses that encode the enzyme reverse transcriptase (RT), enabling them to reverse transcribe their single-stranded viral genomic RNA into double-stranded DNA. This newly synthesized DNA is then integrated into the host cell’s genome, a process that allows the virus to establish a persistent, often lifelong infection. Among the various retroviruses, the human immunodeficiency virus (HIV), the causative agent of acquired immunodeficiency syndrome (AIDS), has consistently been a major focus of scientific research. This is largely due to its high propensity to develop drug resistance, posing significant challenges for treatment and management.

HIV primarily infects CD4+ cells, where its double-stranded DNA (dsDNA) integrates into the cellular genome to form a provirus and replicate. This process damages immune cells through both direct and indirect mechanisms, leading to immunodeficiency and subsequently inducing opportunistic infections and tumors. Research has confirmed that DDX5 plays a key role in the efficient transcription of HIV; it interacts with the transcription trans-activator (Tat) and binds to the hexamethylene bis-acetamide-inducible protein 1 (HEXIM1), thereby enhancing the utilization of positive transcription elongation factor b (P-TEFb) and consequently promoting HIV transcription (Sithole et al., 2020). Furthermore, DDX1 can directly bind to the regulator of virion protein expression (Rev) and Rev response element (RRE); acting as an RNA chaperone, it remodels the structure of the RRE stem-loop II, optimizes Rev binding and oligomerization, and enhances the Rev-RRE nuclear export function, thereby promoting HIV-1 replication (Hammond et al., 2017). Furthermore, DDX3X promotes the initiation of HIV-1 genomic RNA (gRNA) translation and messenger ribonucleoprotein complexes (mRNPs) assembly in an ATP-dependent manner, thereby facilitating HIV-1 replication (Yedavalli et al., 2004; Ishaq et al., 2008; Soto-Rifo et al., 2013; Jansen et al., 2024). Furthermore, studies have confirmed that DDX1 and DDX3X are key factors ensuring the efficient Rev-dependent nuclear export of HIV-1 RNA; DDX3X can colocalize with and bind to the HIV-1 Tat protein within cytoplasmic aggregates, specifically enhancing Tat function (Yasuda-Inoue et al., 2013a). DDX3X can also interact with DDX5 to synergistically enhance Rev function (Yasuda-Inoue et al., 2013b), thereby facilitating HIV replication. Furthermore, HIV-1 can hijack the helicase activity of DDX6 to promote Gag polymerization and capsid assembly at the cell membrane, making DDX6 a key host factor essential for viral capsid assembly (Reed et al., 2012). In the context of the host innate immune response, although DDX3X recognizes abnormal HIV-1 RNA (abortive RNA), HIV can, through specific proteins it encodes, disrupt the structure and activity of MAVS via direct binding, induction of degradation or inhibition of phosphorylation, thereby blocking the antiviral signals mediated by MAVS. Although DDX3X successfully recognizes abnormal viral RNA, the antiviral signal cannot be transmitted downstream; consequently, immune factors such as interferon are not produced efficiently, and the host loses its ability to effectively clear HIV-1, thereby enabling the virus to evade the immune system (Gringhuis et al., 2017). Similarly, in inhibiting viral replication, DDX42 suppresses HIV-1 reverse transcription in a manner independent of the interferon system, thereby reducing the accumulation of viral DNA; it can also block the spread of LINE-1 RNA by binding to it (Bonaventure et al., 2022). Furthermore, HIV can stabilize the viral infectivity factor Vif by downregulating DDX52 levels, thereby sustaining a persistent infection. However, the complex formed by HIV-1 Viral infectivity factor (Vif), DDX52 and apolipoprotein B mRNA editing enzyme catalytic polypeptide-like 3G (APOBEC3G) can downregulate Vif expression via the proteasomal degradation pathway, thereby antagonizing viral infection (Dasgupta et al., 2025).

As a result, host antiviral defenses could be enhanced by interfering with the interaction between DDX5 and HIV, suppressing the activity of the DDX3X enzyme, or increasing the expression of proteins such as DDX42 and DDX52. This paves the way for new therapeutic approaches for the treatment of chronic HIV infection.

## 5 Summary and outlook

As key members of the SF2 helicase family, DDX proteins play a central role in fundamental biological processes such as DNA replication, transcription, and translation. When viruses infect host cells, they often hijack various host factors, including DDX proteins, to facilitate their own replication, induce cellular damage, and evade the host’s innate immune defenses. The core of this process lies in exploiting the host’s RNA metabolism machinery to support viral proliferation and secure a replicative advantage during infection. A systematic examination of the interactions between diverse virus families and DDX proteins—whether through viral hijacking or DDX-mediated mechanisms—reveals that DDX proteins are not simply static antiviral “guardians” or passive “enablers” of viral replication. Instead, they function as dynamic regulators throughout viral infection. DDX proteins can influence viral nucleic acid structure, contribute to the assembly of viral complexes, act as a translation co-factor, and assist in immune evasion; they can also serve as a regulator or initiator of the innate immune response, activating relevant cellular signaling pathways and the interferon response (**Figure 2**; **Table 3**). These complex interactions and mutual constraints position DDX proteins as a “double-edged sword” in the regulation of viral infection: they can both promote viral infection and effectively suppress viral replication. Moreover, due to their inherent enzymatic activity and RNA regulatory capabilities, DDX proteins not only serve as a crucial tool for the host in orchestrating nucleic acid metabolism but also become a prime target for viral exploitation.

**Figure 2:**
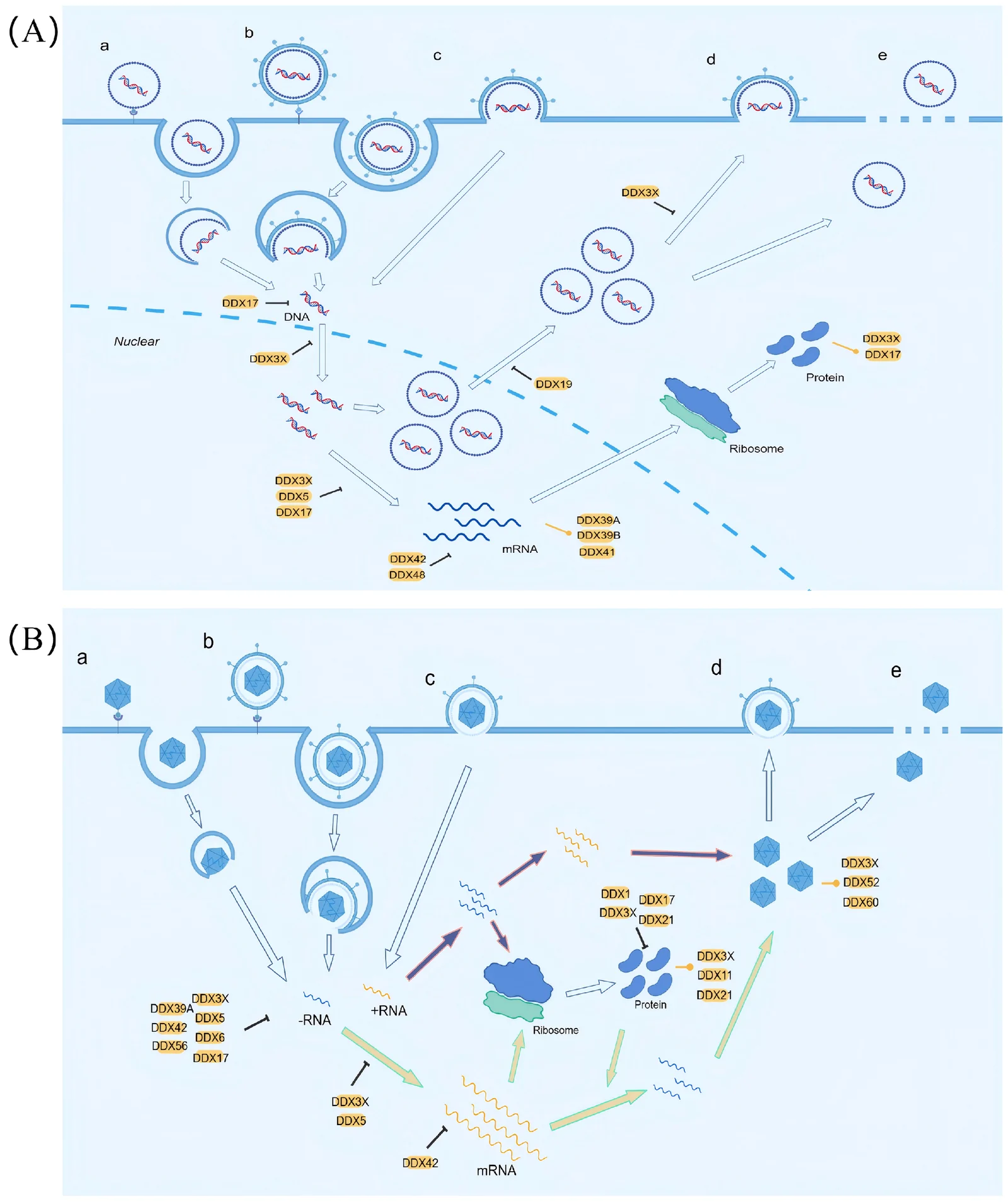
The viral replication process and the sites of interaction with the relevant DDX proteins. **(A)**. The process of DNA virus replication and the sites of involvement of the relevant DDX proteins. **(B)**. The process of RNA virus replication and the sites of involvement of the relevant DDX proteins. [**(a)** Receptor-mediated endocytosis. **(b)** Receptor-mediated membrane fusion. **(c)** Membrane fusion. **(d)** Budding. **(e)** Cell lysis. Orange sections: DDX proteins that can be utilized by viruses. Black sections: DDX proteins that act on viruses.] Created with BioGDP.com.

**Table 3:** Immune signaling pathways involving DDX proteins and their mechanisms.

DDX Protein typing	Relevant immune signaling pathways	Mechanism of action	Outcome of the action	References
DDX5	TLR2/TLR4, NF-κB	Upon infection, DDX5 is recruited to the Hrd1 protein on the ER membrane via a MyD88-dependent pathway, where it is degraded through the ubiquitin-proteasome pathway. This disrupts the DDX5-mediated m6A methylation complex, thereby halting both m6A modification and the subsequent degradation of TLR2/4 mRNA, and promoting the expression of TLR2 and TLR4 and the activation of their downstream NF-κB pathway.	Enhancing the anti-inflammatory response during infection	(Xu et al., 2024)
	RLR	By targeting key components of the RLR signaling pathway (MAVS, MITA, TBK1, IRF3, and IRF7), this compound suppresses the production of type I interferons.	Promotes viral replication	(Zhang et al., 2023)
DDX20	IFN-β	It facilitates the interaction between TBK1 and IRF3, enhances IRF3 phosphorylation induced by external stimuli; this process drives IFN-β expression, which in turn upregulates ISGs such as Cig5 and IFIT1.	Exert an antiviral effect	(Chen et al., 2024)
DDX11	RIG-I-MAVS	Upon specific binding to nucleic acids by RIG-I, the binding affinities of RIG-I for both double-stranded RNA and MAVS are consequently enhanced, thereby regulating RIG-I-MAVS-mediated interferon signaling.	Activates potent interferon signaling and antiviral immunity	(Zhang et al., 2024a)
DDX4	USP7-SOCS1	By binding to the deubiquitinating enzyme ubiquitin-specific protease 7 (USP7), DDX4 disrupts the interaction between USP7 and suppressor of cytokine signaling 1 (SOCS1), leading to the degradation of SOCS1 and thereby enhancing the antiviral function of IFN-I.	Boosting antiviral immunity	(Miao et al., 2024)
DDX54	ALKBH5	The virus modulates the cellular interferon-mediated antiviral response through the activation of the DDX54/m6A/ALKBH5 axis, thereby regulating m6A modifications on specific transcripts.	Negative regulation of the antiviral action of interferon	(Sun et al., 2025)
DDX56	IRF3, IFN-β	By directly targeting the MDA5-mediated signaling triggered by the virus, DDX56 activates KPNA3 and KPNA4 in the signaling cascade, thereby blocking the phosphorylation of IRF3 and its nuclear translocation, thus inhibiting IFN-β production.	Promotes viral replication	(Xu et al., 2022)

Therefore, a comprehensive analysis of the interaction mechanisms between DDX proteins and viruses could provide valuable new insights into the prevention, treatment, and control of viral infections, particularly regarding refractory viruses as well as RNA viruses that exhibit high mutation rates. Although the mechanisms underlying the interactions between certain DDX proteins and viruses have already been clarified, this protein family consists of a large number of members, and many regulatory patterns and viral exploitation strategies remain uncharacterized. Moreover, most current findings are based exclusively on DDX protein-overexpressing or knockout cell lines, and these conclusions have not been validated in animal models or *in vivo* settings; whether unique interaction pathways exist between DDX proteins and viruses is still an open question. Additionally, it remains unclear whether functional interactions occur among different members of the DDX protein family, or whether other DDX proteins can compensate when a particular DDX protein is subverted by viral infection.

Future research should aim to integrate high-throughput omics techniques, such as proteomics and transcriptomics, to systematically identify key DDX proteins associated with different viruses at various stages of their replication cycle, with a particular focus on viruses characterized by high infectivity, broad transmissibility, rapid mutation rates, and control challenges. Concurrently, employing advanced techniques such as single-molecule fluorescence resonance energy transfer (smFRET) and cryo-electron microscopy will allow for a detailed elucidation of the binding dynamics between DDX proteins and viral nucleic acids, the regulatory principles governing ATPase activity, and the molecular basis by which these proteins influence viral proliferation—for instance, through remodeling the viral replication complex (RTC) or modulating host innate immune pathways. This approach will address existing gaps in our understanding of the multi-level interactions among “DDX proteins–viral nucleic acids–host factors”. With the rapid advancement of multi-omics technologies, it is now feasible to construct an “DDX protein–virus–host” interaction network model by integrating multi-dimensional data. Such integration would reveal the regulatory patterns of DDX proteins during viral infection at the systems level, while also exploring potential cooperative relationships among members of the DDX protein family.

In summary, the role of DDX proteins as both regulators of host antiviral defenses and tools utilized by viruses extends beyond mere opposing functions, serving instead as a critical interface of interaction between the host and the virus. As research progresses, our understanding of the interactions and mechanisms between DDX proteins and viruses will become more systematic and in-depth, thereby establishing a robust theoretical framework for developing new, more efficient, and safer antiviral strategies based on these mechanisms.
